# Sulforaphane reduces YAP/∆Np63α signaling to reduce cancer stem cell survival and tumor formation

**DOI:** 10.18632/oncotarget.20562

**Published:** 2017-08-27

**Authors:** Matthew L. Fisher, Nicholas Ciavattone, Daniel Grun, Gautam Adhikary, Richard L. Eckert

**Affiliations:** ^1^ Department of Biochemistry and Molecular Biology, University of Maryland School of Medicine, Baltimore, Maryland, USA; ^2^ Department of Dermatology, University of Maryland School of Medicine, Baltimore, Maryland, USA; ^3^ Department of Reproductive Biology, University of Maryland School of Medicine, Baltimore, Maryland, USA; ^4^ The Marlene and Stewart Greenebaum Comprehensive Cancer Center, University of Maryland School of Medicine, Baltimore, Maryland, USA

**Keywords:** YAP, TAZ, hippo signaling, ∆Np63α, sulforaphane

## Abstract

Epidermal squamous cell carcinoma (SCC) is among the most common cancers. SCC can be treated by surgical excision, but recurrence of therapy-resistant disease is a major problem. We recently showed that YAP1, the Hippo signaling transcription adaptor protein, and ∆Np63α, a key epidermal stem cell survival protein, form a complex to drive epidermal cancer stem cell survival. In the present study, we demonstrate that YAP1 and ∆Np63α are important sulforaphane cancer prevention targets. We show that sulforaphane treatment increases YAP1 phosphorylation and proteolytic degradation. The loss of YAP1 is associated with a reduction in ∆Np63α level and a reduction in ECS cell survival, spheroid formation, invasion and migration. Loss of YAP1 and ∆Np63α is mediated by the proteasome and can be inhibited by lactacystin treatment. YAP1 or ∆Np63α knockdown replicates the responses to sulforaphane, and restoration of YAP1 or ∆Np63α antagonizes sulforaphane action. Sulforaphane suppresses ECS cell tumor formation and this is associated with reduced levels of YAP1 and ∆Np63α. These studies suggest that YAP1 and ∆Np63α may be important sulforaphane cancer preventive targets in epidermal squamous cell carcinoma.

## INTRODUCTION

Epidermal squamous cell carcinoma is an extremely prevalent disease that is caused by skin exposure to various mutagens including UV irradiation [1]. It is treated by surgery, but the recurrence rate approaches 10% and the recurring tumors are aggressive and therapy resistant [1]. Increasing evidence suggests that cancer stem cells have a central role in facilitating tumor growth in squamous cell carcinoma and are important therapy targets [2, 3]. Epidermal squamous cell carcinoma cancer stem cells (ECS cells) express stem cell markers, form aggressive and highly vascularized tumors, and display enhanced migratory and invasive potential [2]. Various proteins have been identified as associated with enhanced ECS cell survival, migration, and tumor formation [2-6]; however, the mechanisms that drive ECS cell survival are not well understood. We recently showed that LATS1, YAP1 and ∆Np63α comprise an important survival cascade in ECS cells [7]. Based on these studies, we proposed that suppression of LATS1 (Hippo) signaling leads to enhanced nuclear accumulation of YAP1 which forms a complex with and stabilizes ∆Np63α to enhance ECS cell survival [7]. Hippo signaling is a centrally important cascade that controls organ growth and limits organ size during development [8]. Large Tumor Suppressor 1 (LATS1), a serine/threonine kinase, is a key regulator in the Hippo signaling cascade [9]. Reduced LATS1 kinase activity is associated with enhanced cell proliferation [9], and LATS1 activity is often constitutively reduced in cancer cells [9]. LATS1 reduces cell proliferation by phosphorylating the pro-proliferation/survival transcription adaptor proteins, YAP1 and TAZ, resulting in their movement to the cytoplasm and subsequent degradation [9]. In contrast, non-phosphorylated YAP1 and TAZ interact in the nucleus to stimulate cell survival and proliferation [10, 11]. YAP1 is overexpressed in many cancers [9] and YAP1 activity is associated with enhanced stem cell survival in epidermis and other tissues [10-12].

∆Np63α is a key member of the p63 family of proteins that control epithelial stem cell status and fate [13, 14]. Studies in mouse epidermis identify ∆Np63α as a key controller of differentiation [13-16]. The function of p63 in epithelial development was shown in p63 knockout mice where the newborn mice die due to an epidermal barrier defect [15]. ∆Np63α is the primary p63 form expressed in squamous epithelial tissues [17] and ∆Np63α overexpression is a frequent event in squamous cell carcinoma [18].

Sulforaphane, 1-isothiocyanato-4-(methylsulfinyl) butane, is a natural isothiocyanate cancer preventive agent derived from broccoli and other cruciferous vegetables [19]. SFN has several desirable properties as a cancer prevention agent, as it is highly bioavailable in blood and tissues, is effective at suppressing tumor growth, and has no known side effects [20-23]. SFN has been shown to inhibit cancer development in various tissues [24-28] including epidermis [6, 29-32], but the molecular mechanism of action is not well understood.

As the LATS1, YAP1, ∆Np63α cascade is a potent driver of cancer stem cell survival [7], we decided to determine whether SFN can suppress activity in this cascade as a mechanism to suppress ECS cell survival. Our studies show that SFN treatment increases YAP1 phosphorylation and degradation, reduces ∆Np63α levels and reduces ECS cell survival, spheroid formation, invasion, migration and tumor formation.

## RESULTS

### SFN impacts YAP1 signaling

**Figure 1:**
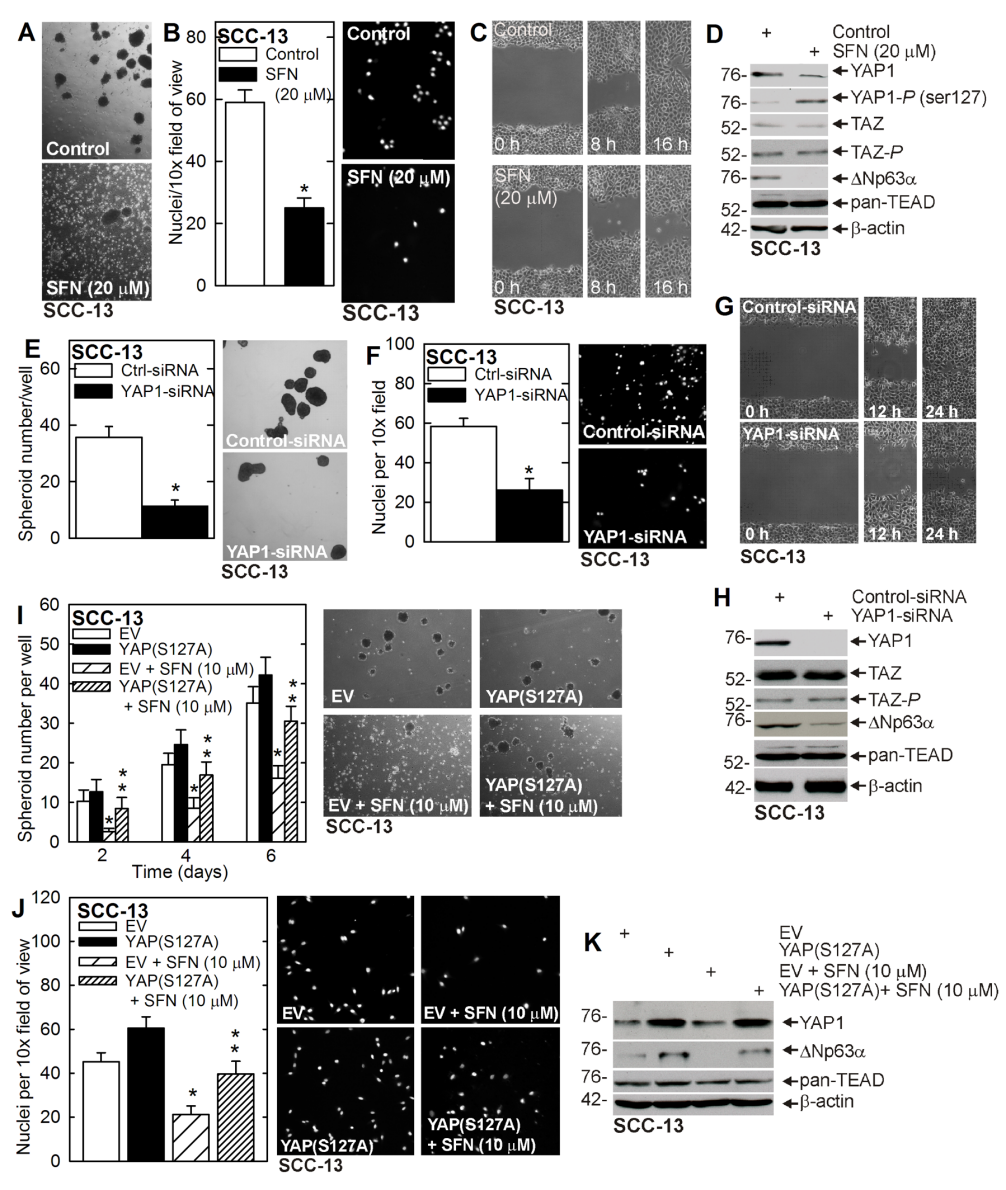
Sulforaphane targets YAP1/∆Np63α to suppress ECS cell phenotype **A. B. C.** ECS cells were grown for 8 d as spheroids and treated with 0 or 20 μM SFN for 48 h before image acquisition. ECS cells were seeded on a matrigel-coated membrane in a Millicell chamber for invasion assay and then treated with 0 or 20 μM SFN for 20 h. ECS cells were plated as high density confluent monolayers for wound closure assay in the presence of 0 or 20 μM SFN. The values are mean ± SEM and the asterisks indicate a significant reduction (*n* = 3, *p* < 0.005). **D.** SFN treatment reduces YAP1, increases YAP1-*P* and reduces ∆Np63α. Cells were grown as spheroids for 8 d, treated with 0 or 20 μM SFN for 48 h and lysates were collected for immunoblot. **E. F. G.** SCC-13 cells were electroporated with control- or YAP1-siRNA and plated for spheroid formation, invasion and migration assay. The values are mean ± SEM and the asterisks indicate a significant reduction (*n* = 3, *p* < 0.005). **H.** YAP1-siRNA treatment reduces YAP1 and ∆Np63α level, but does not impact TAZ or TEAD levels. **I. J.** SCC-13 cells, electroporated with empty vector (EV) or YAP(S127A), were seeded for spheroid growth or invasion assay in the presence of 0 or 10 μM SFN. Spheroid number was monitored at 6 d. The single asterisk indicates a significant reduction in SFN treated as compared to untreated control cultures. The double asterisks indicate a significant increase as compared to the SFN treated group (*n* = 3, *p* < 0.01). **K**. Immunoblot of extracts prepared from 5 d spheroid cultures (panel I).

A small population of squamous cell carcinoma cells (0.15%) survive and grow as spheroids in non-attached conditions, and these ECS cells display elevated levels of epidermal and embryonic stem cell markers, and enhanced ability to invade matrigel and migrate [2, 4]. Moreover, ECS cells form highly aggressive and vascularized tumors as compared to non-stem cancer cells [2, 3]. In the present study we examine the impact of SFN treatment on ECS cells [6, 32]. SFN treatment of ECS cell spheroid cultures reduces spheroid formation and enhances spheroid fragmentation (Figure 1A) and also suppresses ECS cell matrigel invasion (Figure 1B) and migration on plastic (Figure 1C). We recently showed that a signaling cascade that involves YAP1 and ∆Np63α plays an important role in ECS cell survival [7]. We therefore assessed the impact of SFN on the YAP1, TAZ, ∆Np63α and TEAD transcription factors. TEAD factors comprise a family of four transcription factors that are frequent targets of the YAP1 and TAZ transcriptional adaptors [9]. As shown in Figure 1D, SFN treatment reduces YAP1 and increases YAP1-*P*, and this is associated with reduced ∆Np63α. In contrast, TAZ, TAZ-*P* and TEAD levels are not altered.

The finding that YAP1 phosphorylation is altered by SFN treatment prompted us to examine the impact of YAP1 knockdown on ECS cell survival. Figure 1E/1F/1G shows that YAP1 knockdown reduces spheroid formation, matrigel invasion and migration. Figure 1H confirms YAP1-siRNA dependent YAP1 knockdown and loss of ∆Np63α, and confirm no change in TAZ, TAZ-*P* or TEAD factor level. To confirm that YAP1 is a relevant SFN target, we examined the impact of constitutively-active YAP1 expression on SFN suppression of ECS cell spheroid formation and invasion. Figure 1I/1J shows that YAP(S127A) expression partially reverses SFN suppression of spheroid formation and invasion, confirming YAP1 loss is essential for SFN action. Figure 1K shows that YAP(S127A) expression is associated with increased ∆Np63α expression, which is consistent with a role for YAP1 in stabilizing ∆Np63α [7].

### Role of ∆Np63α

**Figure 2:**
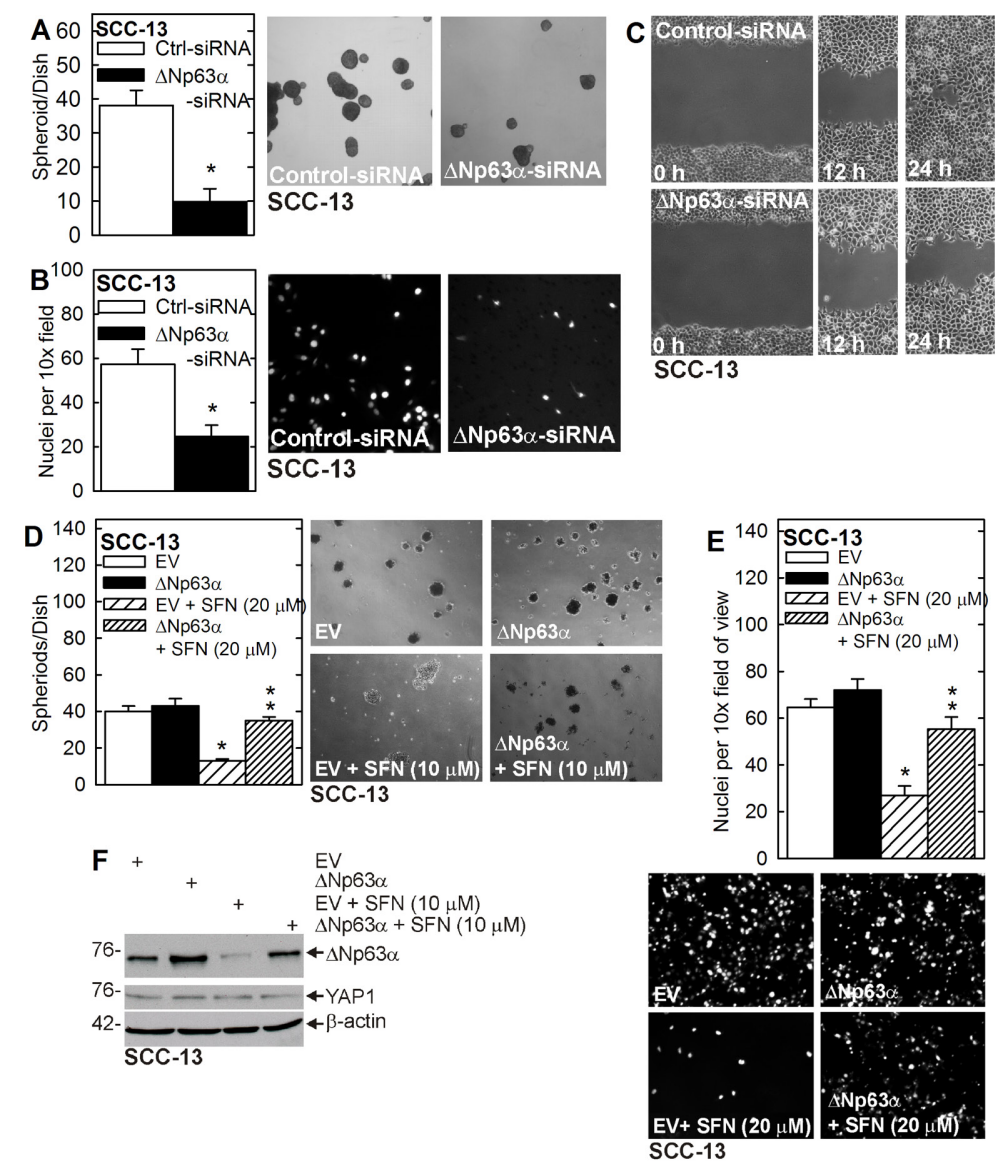
∆Np63α drives the ECS cell phenotype **A. B. C.** SCC-13 cells were double electroporated with control- or ∆Np63α-siRNA and seeded for spheroid formation, invasion and migration assay. The values are mean ± SEM and the asterisks indicate a significant reduction (*n* = 3, *p* < 0.01). **D. E.** ECS cells were electroporated with empty vector (EV) or ∆Np63α expression vector, and seeded for spheroid formation and invasion assays in the presence of 0 or 20 μM SFN. The single asterisk indicates a significant reduction in SFN treated as compared to untreated control cultures. The double asterisks indicate a significant increase as compared to the SFN treated group (*n* = 3, *p* < 0.01). **F.** Cells, treated as indicated, were grown in non-attached conditions for 5 days and lysates were prepared for immunoblot.

We have reported that YAP1 acts to maintain ∆Np63α level and that ∆Np63α is required for ECS cell survival, spheroid formation and invasion [7]. Figure 1D shows that SFN treatment reduces ∆Np63α level. To determine whether loss of ∆Np63α is required for SFN action, we monitored the impact of ∆Np63α knockdown on ECS cell function, and determined that ∆Np63α overexpression can reverse SFN action. Figure 2A/2B/2C shows that loss of ∆Np63α reduces ECS cell spheroid formation, invasion and migration. Figure 2D/2E shows that SFN treatment reduces ECS cell spheroid formation and invasion and that these changes are reversed by ∆Np63α overexpression. Figure 2F confirms overexpression of ∆Np63α and expression vector treated cells and shows that increasing ∆Np63α does not impact YAP1 level.

**Figure 3:**
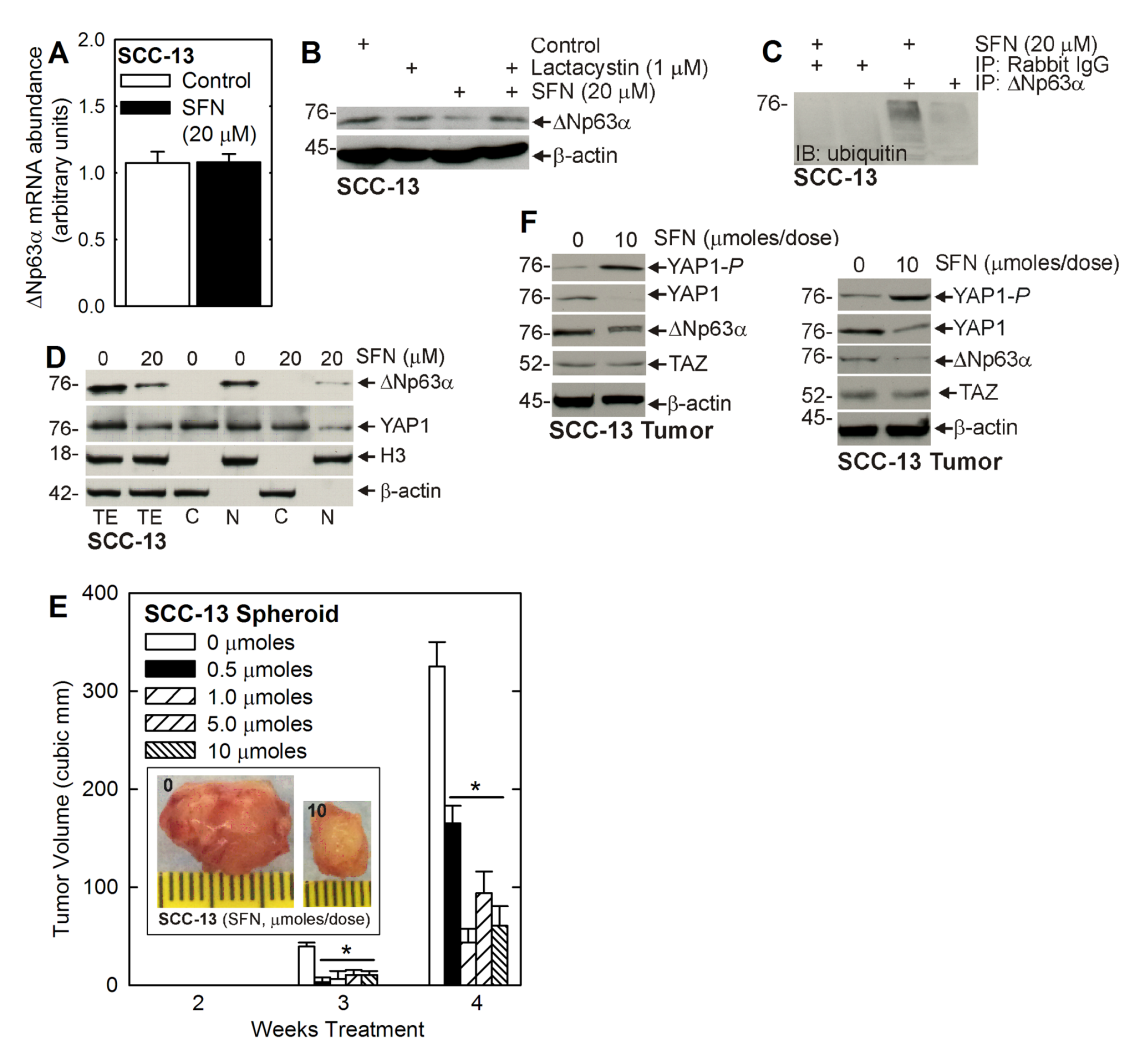
SFN induced proteasome-dependent loss of ∆Np63α **A.** ECS cells were treated with 0 or 20 μM SFN for 48 h and extracts were prepared for assay of ∆Np63α mRNA by qRT-PCR. **B.** ECS cells were pre-treated with 1 μM lactacystin for 1 h, prior to the addition of 20 μM SFN for 24 h. **C.** ECS cells were treated with SFN for 48 h and lysates immunoprecipitated with anti-∆Np63α for anti-ubiquitin immunoblot. **D.** Equal cell equivalents of total (TE), nuclear (N), and cytosolic (C) extract, prepared from control or 48 h SFN treated ECS cells, were electrophoresed for immunoblot detection of ∆Np63α, YAP1, histone 3 (nuclear marker) and β-actin (cytoplasmic marker). **E.** ECS cells (100,000 cells derived from SCC-13) were injected into each front flank in NSG mice. Beginning at 1 d post-injection, SFN was delivered by gavage, three times per week on alternate days at the indicated number of micromoles/dose. Images represent appearance and size of typical control and SFN-treated 4 wk tumors. The values are mean ± SEM and asterisks indicate significant change compared to control, *n* = 5 mice (10 tumors), *p* < 0.01. **F** Tumors were harvested at 4 wk and extracts were prepared for immunoblot. Blots are shown from two representative tumors.

To understand the mechanism of ∆Np63α reduction, we monitored the impact of SFN treatment on ∆Np63α mRNA and found no change (Figure 3A). We then examined the role of the proteasome. ECS cells were treated with SFN in the presence or absence of lactacystin, a proteasome inhibitor. Figure 3B shows that SFN treatment reduces ∆Np63α level and that this is reversed by co-treatment with lactacystin. Moreover, SFN treatment is associated with enhanced ubiquitination of ∆Np63α, which is consistent with proteasome-associated degradation (Figure 3C). Thus, SFN stimulated ∆Np63α turnover is proteasome-mediated. Figure 3D indicates that most of cellular ∆Np63α is in the nucleus, as is 50% of YAP1, and that the nuclear level of both proteins is reduced by treatment with SFN.

### SFN impact on tumor formation

We next determined the impact of SFN treatment on tumor formation. Figure 3E shows that SFN treatment produces a dose-dependent reduction in tumor formation that is optimal at 0.5 to 1 micromoles/dose. Figure 3F shows immunoblots of extract prepared from two representative tumors showing that SFN treatment is associated with reduced levels of YAP1 and ∆Np63α, and increased YAP1-*P* formation. In contrast, TAZ levels are not altered by SFN treatment (Figure 3F).

### Role of YAP1 and SFN in HaCaT cells

**Figure 4:**
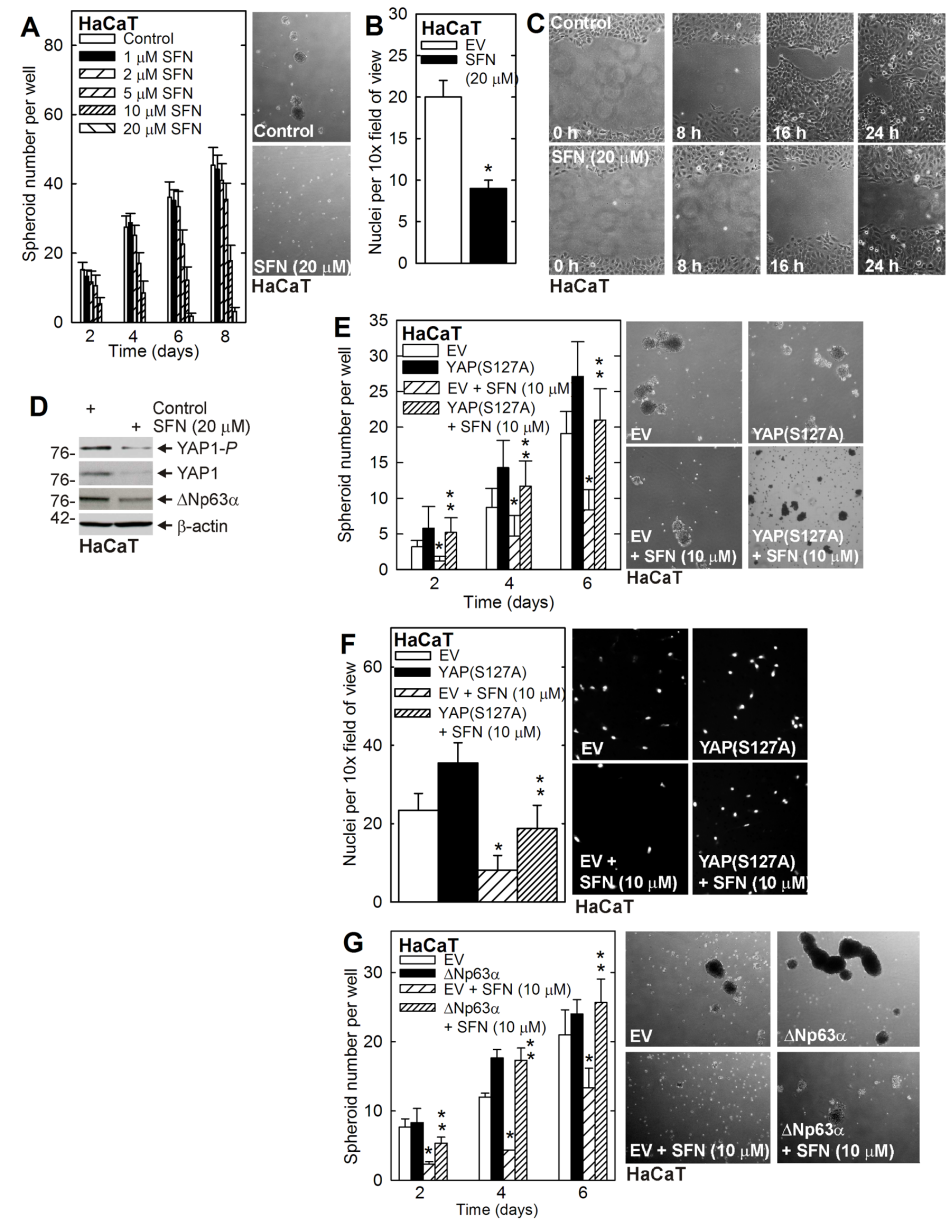
YAP1 and ∆Np63α and the HaCaT cell response to SFN **A. B. C.** HaCaT cells were seeded for spheroid formation, invasion and migration assay. Spheroids were counted and photographed at 8 d. The values are mean ± SEM. A significant reduction was in spheroid number was observed at 4, 6 and 8 d at 10 and 20 μM SFN (*n* = 3, *p* < 0.005). **D.** Cells were grown as spheroids for 8 d and then treated with 20 μM SFN for 48 h prior to collection of lysates for immunoblot. **E. F.** HaCaT cells were electroporated with empty vector (EV) or YAP(S127A) expression vector and at 24 h post-electroporation were seeded for spheroid formation and invasion assay in the presence of 0 or 20 μM SFN. The image shows 6 d spheroids. **G.** HaCaT cells were electroporated as indicated and then seeded for spheroid growth assay in the presence of 0 or 10 μM SFN. The images show 6 d spheroids. The values are mean ± SEM. The single asterisk indicates a significant reduction in SFN treated versus untreated control cultures. The double asterisks indicate a significant increase as compared to the SFN treated group (*n* = 3, *p* < 0.005).

The above studies indicate that SFN reduces YAP1 and ∆Np63α level to reduce survival of SCC-13 derived ECS cells. To determine whether this is a general property shared among epidermis-derived cells, we examined SFN regulation of YAP1 and ∆Np63α in HaCaT cells. As shown in Figure 4A/4B/4C, SFN treatment of HaCaT cell-derived ECS cells reduces spheroid formation, matrigel invasion and migration. Figure 4D shows that SFN treatment reduces YAP1 and ∆Np63α. Expression of YAP(S127A) reverses SFN suppression of spheroid formation and matrigel invasion (Figure 4E/4F). Moreover, ∆Np63α overexpression reverses SFN suppression of spheroid formation (Figure 4G).

### Role of TAZ in response to SFN treatment

**Figure 5:**
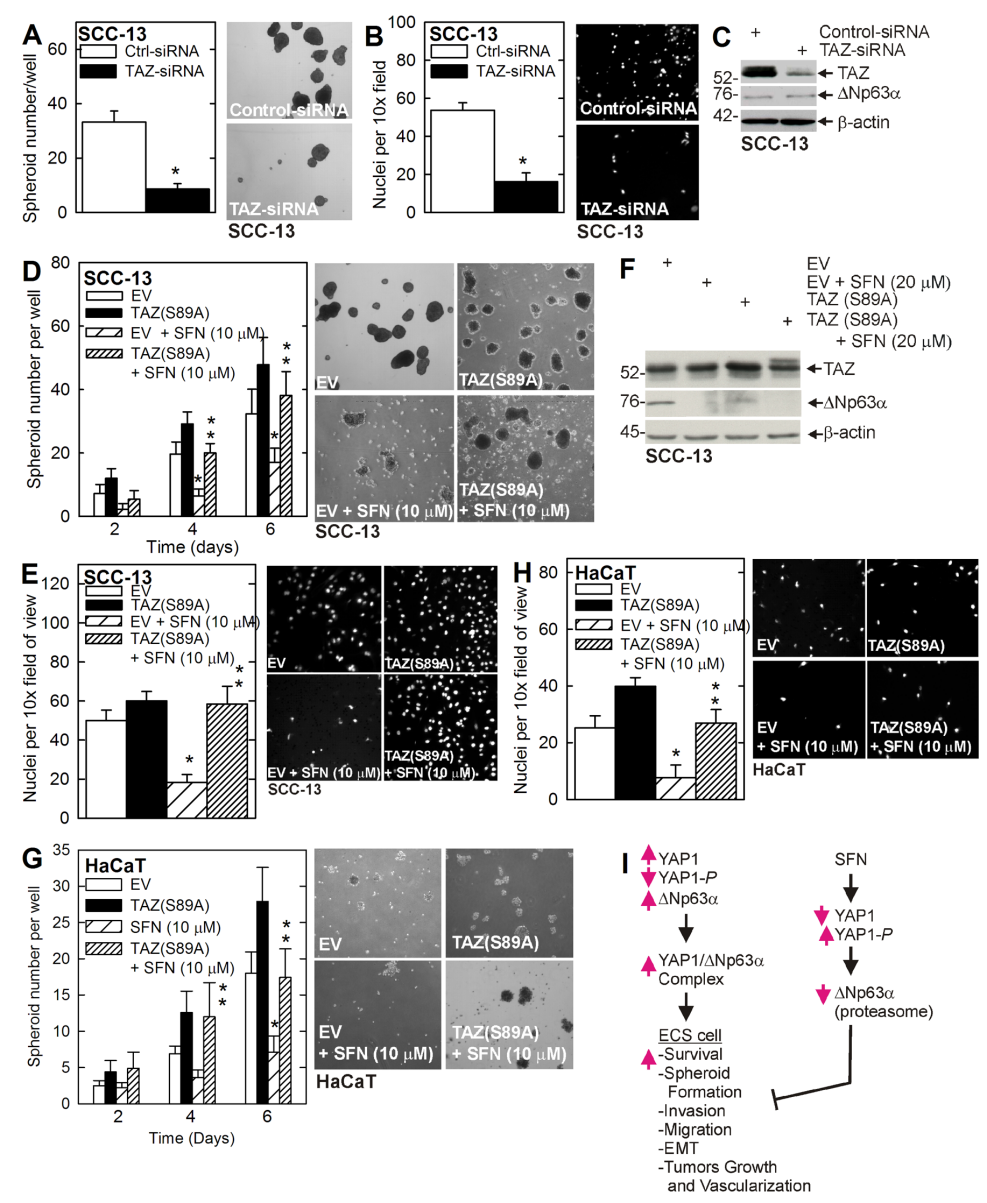
TAZ stimulates the ECS cell phenotype **A. B. C.** ECS cells were electroporated with control- or TAZ-siRNA and then seeded for spheroid formation and 18 h invasion assay. Extracts for immunoblot were prepared at 48 h post-electroporation. The images are 6 d spheroids. The values are mean + SEM, and the asterisk indicates a significant reduction in SFN treated versus untreated control cultures (*n* = 3, *p* < 0.005). **D. E. F.** SCC-13 cells were electroporated as indicated and the cells were seeded for spheroid formation and 18 h invasion assay in the presence of 0 or 10 μM SFN. The images are of 6 d spheroids. The single asterisk indicates a significant reduction in SFN treated versus untreated control cultures. The double asterisks indicate a significant increase as compared to the SFN treated group (*n* = 3, *p* < 0.005). Extracts were prepared from 6 d spheroids for immunoblot. **G**. **H**. HaCaT cells were electroporated with EV or TAZ(S89A) and then seeded for spheroid and invasion assay with or without 10 μM SFN. The asterisks indicate significance as in panels **I**. Model of SFN action. YAP1 levels are elevated in ECS cells where it binds to ∆Np63α leading to elevated ∆Np63α levels which drives ECS cell survival, etc. SFN treatment stimulates YAP1 phosphorylation leading to reduced YAP1 level leading to proteasome-dependent loss of ∆Np63α which results in reduced ECS cell survival and reduced tumor growth.

The YAP1/TAZ transcription adaptor proteins are important controllers of cancer cell survival [9]. Our studies show that SFN treatment of cultured ECS cells (Figure 1D), or ECS cell derived tumors (Figure 3F), reduces YAP1 and ∆Np63α level, but does not alter TAZ level, suggesting that TAZ may not be a mediator of SFN action. However, we wanted to determine whether TAZ can influence the ECS cell response to SFN. Figure 5A/5B shows that TAZ knockdown reduces ECS cell spheroid formation and invasion but that loss of TAZ expression is not associated with a reduction in ∆Np63α level (Figure 5C). Figure 5D/5E shows that expression of constitutively-active TAZ, TAZ(S89A), reverses the SFN suppression of spheroid formation and matrigel invasion. Figure 5F demonstrates that TAZ(S89A) expression slightly increases ∆Np63α level. To determine whether this is a general effect, we assessed the role of TAZ in HaCaT cells. Figure 5G/5H show that expression of TAZ(S89A) can partially reverse SFN suppression of HaCaT cell spheroid formation and matrigel invasion.

## DISCUSSION

We recently showed that LATS1, YAP1 and ∆Np63α are part of an important ECS cell survival cascade [7]. In ECS cells, reduced LATS1 (Hippo) signaling leads to reduce YAP1 phosphorylation and enhanced nuclear accumulation of non-phosphorylated YAP1 which interacts with and stabilizes ∆Np63α to drive survival signaling [7]. ∆Np63α is a key member of the p63 family of proteins which is required for normal stem cell survival and differentiation in epidermis [13, 14]. Moreover, YAP1 and ∆Np63α have important roles in cancer [9]. We have shown that YAP1 and ∆Np63∆ are overexpressed in squamous cell carcinoma and the level of these pro-survival proteins is further markedly enriched in ECS cells. ECS cells comprise a small subpopulation (0.15%) of the total tumor cell population [2] and enriched ECS cells form highly aggressive, rapidly growing and highly vascularized and invasive tumors [2, 3]. In addition, loss of YAP1 expression leads to reduced ECS cell survival and inhibition of YAP1 function reduces tumor formation [7]. Thus, targeting YAP1 and ∆Np63α is an important potential strategy for reducing cancer stem cell survival in squamous cell carcinoma.

The idea that cancer chemoprevention agents may reduce survival of cancer stem cells is an important and evolving concept. Recent studies in colon and breast cancer suggest that diet-derived prevention agents can selectively target cancer stem cells [33, 34]. An interesting and important observation is that cancer stem cells can be more sensitive to dietary preventive agents than non-stem cancer cells [34]. However, the mechanisms that confer this sensitivity are not well understood. SFN is an important cruciferous vegetable-derived (broccoli, etc.) cancer prevention agent [35] that has high bioavailability *in vivo* [34, 36] and displays efficacy against skin cancer in several model systems [31, 36, 37]. Moreover, it can be detected at bioactive levels in blood and tissues of broccoli-consuming human patients showing that biologically relevant levels can be achieved [31]. The concentrations of SFN used in the present studies are equivalent to levels that produce biological responses in humans.

We test the hypothesis that YAP1/∆Np63α signaling is targeted and suppressed by SFN as a mechanism of cancer prevention/therapy. YAP1 is resident in the nucleus where it activates cell survival signaling and proliferation. In contrast, phosphorylated YAP1 is excluded from the nucleus and subject to proteasome-mediated degradation [9, 38, 39]. Our studies show that SFN treatment increases YAP1-*P* and reduces YAP1, and that this is associated with SFN suppression of cell spheroid formation, invasion and migration. Consistent with YAP1 functioning as a SFN target, expression of YAP(S127A), a constitutively active form of YAP1, reverses the SFN-dependent reduction in ECS cell survival, spheroid formation, matrigel invasion and migration. This suggests that YAP1 inactivation is required for SFN suppression of the ECS cell phenotype. This is consistent with recent findings suggesting that YAP1 is a key anticancer target [9, 38, 39].

Moreover, loss of YAP1 leads to a reduction in ∆Np63α. This is an important event, as SFN action can be reversed by forced expression of ∆Np63α in SFN challenged cultures. Moreover, ∆Np63α knockdown reduces ECS cell spheroid formation, matrigel invasion and migration. This suggests that ∆Np63α is required for YAP1-induced survival in ECS cells. To understand the mechanism of regulation of ∆Np63α level by YAP1, ECS cells were treated with SFN and ∆Np63α mRNA and protein levels were monitored. SFN treatment does not impact ∆Np63α mRNA level, but a large drop in ∆Np63α level is observed. This suggests that the regulation is not at the level of transcription or mRNA stability. Treatment of ECS cells with SFN in the presence of proteasome inhibitor restores ∆Np63α expression. This suggests a model in which YAP1/∆Np63α interaction stabilizes nuclear ∆Np63α leading to enhanced survival signaling.

To assess whether loss of YAP1/∆Np63α is a common response to SFN treatment, we examined the impact of SFN treatment in HaCaT cell-derived ECS cells. HaCaT cells are an immortalized line of epidermis-derived keratinocytes [40]. The HaCaT studies confirm that SFN treatment reduces spheroid formation, matrigel invasion and migration, and show that this is associated with reduced YAP1 and reduced ∆Np63α. Moreover, forced expression of YAP(S127A) or ∆Np63α in SFN treated HaCaT-derived ECS cells, protects the cells against SFN and restores ECS cell spheroid formation, and matrigel invasion. Thus, SFN regulation of YAP1 and ∆Np63α is observed in multiple epidermis-derived cell types.

To assess the role of these signaling proteins during tumor formation and response to SFN, we treated ECS cell tumor xenografts with SFN and monitored the impact on YAP1/∆Np63α level. These studies show that SFN reduces ECS cell tumor formation and that this is associated with increased YAP1-*P*, reduced total YAP1 and reduced ∆Np63α. These findings suggest that the SFN-stimulated signaling changes observed in cultured cells are also observed in SFN treated tumors *in vivo*.

YAP1 often interacts with TAZ to modulate transcription [38, 39]. Our previous study shows that YAP1 is an important mediator of ECS cell survival, but that TAZ is not required [7]. Indeed, our present study shows that SFN treatment does not reduced TAZ level in cultured cells or in SFN-treated tumors, suggesting that TAZ does not play a major role in the response to SFN. However, we wanted to determine whether TAZ can influence ECS cell function. These studies reveal that TAZ loss reduces spheroid formation and matrigel invasion, but that loss of TAZ is not associated with loss of ∆Np63α. Moreover, forced expression of TAZ(S89A), a constitutively activate form of TAZ, antagonizes SFN action and restores spheroid formation and matrigel invasion. We conclude that although TAZ does not appear to play a role in SFN suppression of the ECS cell phenotype, TAZ can independently impact ECS cell survival and resistance to SFN. Additional studies will be necessary to further understand the mechanism of TAZ action in this context.

Based on these studies we propose that ECS cell survival is associated with elevated YAP1 (and reduced YAP1-*P*) leading to YAP1 association of with and stabilization of ∆Np63α, and that ∆Np63α then drives an increase in ECS cell survival (Figure 5I). We further propose that SFN treatment increases YAP1-*P* and reduces YAP1 level, and that loss of YAP1 leads to proteolytic degradation of ∆Np63α to reduce ECS cell survival and growth. Our observations suggest that this mechanism exists in multiple cell types and in tumors, and that this mechanism is worthy of additional study as a potentially important mechanism of SFN-mediated cancer prevention and therapy.

## MATERIALS AND METHODS

### Antibodies and reagents

Sodium pyruvate (11360-070), DMEM (11960-077), 0.25% trypsin-EDTA (25200-056) and L-Glutamine (25030-164) were purchased from Gibco (Grand Island, NY). Heat-inactivated fetal calf serum (FCS, F4135), lactacystin (L6785) and anti-β-actin (A5441) were purchased from Sigma (St. Louis, Mo). Cell lysis Buffer (9803) was purchased from Cell Signaling Technology (Danvers, MA). YAP1 (4912), YAP1-*P* (13008), Histone 3 (#9717) and TAZ (4883) antibodies were purchased from Cell Signaling Technologies. Anti-p63 (sc-8431), anti-ubiquitin (sc-9133) and anti-TAZ-*P* (17610) were purchased from Santa Cruz. Anti-pan-TEAD (ab1791) was purchased from Abcam (Cambridge, MA). Peroxidase-conjugated anti-mouse IgG (NXA931) and anti-rabbit IgG (NA934V) were obtained from GE healthcare (Buckinghamshire, UK). DAPI (D9542) was purchased from Sigma Chemicals (St. Louis, MO). p63- (sc-36161), TAZ- (sc-38568A) and control-siRNA (sc-37007) were purchased from Santa Cruz (Dallas, TX). YAP1-siRNA (S102662954) was purchased from Qiagen (Valencia, CA). Matrigel (354234) and BD Biocoat cell inserts (353097) were purchased from BD Biosciences (Franklin Lakes, NJ). Sulforaphane (S8044) was purchased from LKT Laboratories INC (St. Paul, MN). YAP(S127A) (Addgene plasmid # 27370) and TAZ(S89A) (Addgene plasmid # 32840) were from Kunliang Guan [41].

### Immunoblot

Cell extracts were prepared in lysis buffer, and equivalent amounts of protein were electrophoresed on denaturing and reducing 10% polyacrylamide gels and transferred to nitrocellulose membrane. Membranes were blocked with 5% nonfat dry milk for one hour and incubated in 5% nonfat dry milk with containing 1:1000 diluted primary antibody. Blots were washed and then incubated with secondary antibody (1:5000) for 2 h. Secondary antibody binding was visualized using ECL (Amersham) chemiluminescence detection technology.

### Spheroid formation assay

Cancer cells were maintained under attached conditions in growth media containing DMEM (Invitrogen, Frederick, MD) supplemented with 4.5 mg/ml D-glucose, 2 μM L-glutamine, 100 mM sodium pyruvate, and 5% fetal calf serum. For spheroid formation, near-confluent monolayer cultures are dissociated with 0.25% trypsin, followed by serum-dependent trypsin inactivation. The cells are collected by centrifugation, and resuspended in spheroid media, consisting of DMEM/F12 (1:1) (DMT-10-090-CV, Mediatech INC, Manassa, VA) containing 2% B27 serum-free supplement (17504-044, Invitrogen, Frederick, MD), 20 ng/ml EGF (E4269, Sigma, St. Louis), 0.4% bovine serum albumin (B4287, Sigma) and 4 μg/ml insulin (19278 Sigma, St. Louis, MO.), and plated at 40,000 cells per 9.6 cm^2^ well in six well ultra-low attachment Costar cluster dishes (4371, Corning, Tewksbury, MA).

### Cell fractionation studies

The cells used for this experiment were day 8 spheroids that were treated with 0 or 20 μM SFN for 48 h before extract preparation. The NE-PER Nuclear and Cytoplasmic Extraction Kit (product # 78833) was obtained from Thermo Scientific (Waltham, MA). For total extracts, 40 μg of protein was electrophoresed and equal numbers of cell equivalents were loaded in lanes comparing the cytosol and nuclear fractions.

### qRT-PCR

Total RNA was isolated using the RNAspin Mini Kit (GE Healthcare) and reverse transcribed using the Superscript III reverse transcriptase (Invitrogen, Carlsbad, CA). RNA (1 μg) was used for cDNA preparation. The Light Cycler 480 SYBR Green I Master mix (Roche Diagnostics) was used to measure mRNA level. ∆Np63α mRNAlevel was detected and signals were normalized to the level of cyclophilin A mRNA. The following gene specific primers were used for detection of mRNA levels: ∆Np63α (forward: 5’-5‘-GGA AAA CAA TGC CCA GAC TCA, reverse: 5’-5’-TGT TCA GGA GCC CCA GGT T) and cyclophilin A (forward: 5’-CAT CTG CAC TGC CAA GAC TGA, reverse: 5’-TTC ATG CCT TCT TTC ACT TTGC).

### Electroporation of nucleic acids

Cancer cells (150,000) were plated in 60 mm plates in growth media. After 24 h, when approximately 50% confluent, the cells were collected using 0.25% trypsin, centrifuged at 200 x g, washed with sterile phosphate-buffered saline (pH 7.5), and suspended in 100 μl of keratinocyte nucleofection reagent VPD-1002 (Walkersville, MD) for electroporation with siRNA or plasmid. The cell suspension, contained either 3 µg of siRNA or 2 µg of plasmid was gently mixed and electroporated using the T-018 setting on the AMAXA Electroporator. Immediately after electroporation, pre-warmed spheroid media was added and the suspension was transferred to monolayer culture. After 24 h, the cells were harvested and plated for spheroid formation, migration and invasion assays. For siRNA experiments, the cells were harvested and electroporated a second time, following the same protocol, 72 h after the initial electroporation.

### Invasion and migration assays

Matrigel (BD Biolabs) was diluted in 0.01 M Tris-HCl/0.7% NaCl and filter sterilized and 0.1 ml was used to cover BD BioCoat cell inserts. After 2 h, cells were harvested and 25,000 cells were plated in 100 μl of growth medium containing 1% FCS on top of the Matrigel. Growth medium containing 10% FCS was added to the lower well chamber and the cells were incubated overnight at 37 C. The following day, excess cells from the top side of the membrane were removed with a cotton swab, and the membrane was rinsed with phosphate buffered saline, fixed with 4% paraformaldehyde for 10 min, washed, and stained in 1 μg/ml DAPI for 10 min. The underside of the membrane was photographed with an inverted fluorescent microscope to count the number of nuclei. For migration, SCC-13 cells (2 million) were plated on 10 cm dishes in spheroid media under monolayer conditions and allowed to attach overnight. Once confluent, a 10 μl pipette was used to create scratch wounds. The dishes were washed with phosphate buffered saline to remove the dislodged cells and fresh spheroid medium was added with or without SFN. Images were taken at 10x to monitor cell migration into the wounded area.

### Tumor xenograft assays

Spheroid-derived cancer cells were prepared as a single cell suspension by trypsin digestion, resuspended in phosphate buffered saline containing 30% Matrigel and 100 μl containing 0.5 x 10^6^ cells was injected subcutaneously in the two front flanks of NOD *scid* IL2 receptor gamma chain knockout mice (NSG mice) using a 26.5 gauge needle. Five mice were used per group (two tumors per mouse). SFN was dissolved in sterile saline and delivered by oral gavage at 20 micromoles per dose in 100 μL on alternate days (M/W/F). Tumor growth was monitored by measuring tumor diameter and calculating tumor volume using the formula, volume = 4/3π x (diameter/2)^3^. Mice were euthanized by injection of 250 μl of a 2.5% stock of Avertin per mouse followed by cervical dislocation of the neck. Tumor samples were harvested to prepare extracts for immunoblot and sections for immunostaining. These experiments were reviewed and approved by the University of Maryland-Baltimore Institutional Animal Care and Use Committee.
